# 2D material inorganic liquid crystals for tunable deep UV light modulation

**DOI:** 10.1093/nsr/nwac252

**Published:** 2022-11-09

**Authors:** Young Hee Lee

**Affiliations:** IBS Center for Integrated Nanostructure Physics, Institute for Basic Science, Sungkyunkwan University, South Korea; Department of Energy Science and Department of Physics, Sungkyunkwan University, South Korea

Modulation of light refers to the modulation of electromagnetic radiation in the optical region including visible light as well as ultraviolet and infrared radiation, in which the amplitude (and therefore the intensity), phase, frequency or polarization of the radiated oscillations can be changed [1]. Optical modulator devices are widely used in industry, as represented by liquid crystal optical modulators having an annual global market worth of over US$100 billion. Currently, optical modulators working in the visible and infrared regions are mature, while those that can work in the deep ultraviolet (UV) region are rare. It is difficult for existing technologies like organic liquid crystal optical modulators to modulate deep UV light because they use organic molecules as active materials, which absorb UV heavily and/or are not stable under high energetic deep UV photons. On the other hand, inorganic birefringent crystals can modulate deep UV light while it has fixed birefringence after fabrication and cannot be tuned by an external field [2]. Therefore, the community still lacks a technology that can stably and continuously tune deep UV light in a transmissive manner.

Recently, joint research [3] led by Bilu Liu of Tsinghua University, and Hui-Ming Cheng and Baofu Ding of the Chinese Academy of Sciences, involved the fabrication of a stable and magnetic-tunable deep UV light modulator using two-dimensional (2D) hexagonal boron nitride (h-BN) based inorganic liquid crystals, addressing the lack of transmissive light modulators in this deep UV region.

The authors first produced 2D h-BN flakes via a top-down exfoliation technique; these have an average flake size and thickness of 477 nm and 7.5 nm, respectively. The h-BN flakes, dispersed in aqueous solution, behave as inorganic liquid crystals. The 2D h-BN has a large optical bandgap of ∼6 eV, guaranteeing its high transparency at short wavelengths, i.e. >70% transmittance at a wavelength of 266 nm. In addition, the 2D h-BN flakes are found to show anisotropic magnetism with the in-plane easy axis and the out-of-plane hard axis, that is, the alignment of h-BN flakes in dispersion can be tuned by an external magnetic field. Such magnetic field included alignment of 2D flakes can introduce birefringence to the system through a magneto-optical effect, serving as the foundation for light modulation [4]. Surprisingly, the 2D h-BN is found to have an extremely large optical anisotropy factor and a resultant specific magneto-optical Cotton–Mouton coefficient which is five orders of magnitude larger than other deep UV transparent media.

Thanks to its wide bandgap, high stability under deep UV irradiation and extremely sensitive magneto-optical response, they further fabricated a light modulator using 2D h-BN inorganic liquid crystals that can tune deep UV light down to 266 nm, entering the deep UV-C region. Such a 2D h-BN liquid crystal light modulator can tune deep UV light with a phase retardation in the range of (0–1/8)π, and is found to be stable after repeated operation.

**Figure 1.**
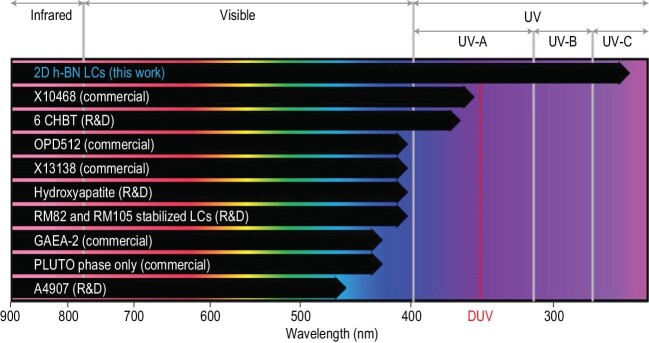
Liquid-crystal-based optical modulators. Comparison of commercialized and research-and-development (R&D) stage liquid-crystal-based optical modulators with proven stability in the denoted spectral range. The 2D h-BN inorganic liquid crystal optical modulator can stably and continuously tune deep UV-C light down to 266 nm. Reprinted from Ref. [3] with permission from Nature Publication Group.

This milestone study is a breakthrough as it is the first time that h-BN liquid crystal devices have been used to tune deep UV light (Fig. 1) [3], creating numerous exciting opportunities ahead. It is not difficult to imagine that the widely used birefringence-tunable optics can be transferred from current visible and infrared regions to the technologically important deep UV region. Meanwhile, considering that 2D materials can be massively produced by top-down exfoliation methods [5–7] or bottom-up growth methods [8], it is reasonable to infer that 2D-material-based inorganic liquid crystal devices can be scalably manufactured with low cost, as demonstrated in recent work using 2D mineral material vermiculite [9].
